# A robust deep learning approach for impulse noise filtering using hybrid auto-encoder with fuzzy median filter

**DOI:** 10.1371/journal.pone.0343141

**Published:** 2026-04-03

**Authors:** Muhammad Naeem, Sohail Masood Bhatti, Muhammad Rashid, Arfan Jaffar, Sheeraz Akram, Benish Fida, Awais Ahmad

**Affiliations:** 1 Faculty of Computer Science & Information Technology, The Superior University, Lahore, Pakistan; 2 Intelligent Data Visual Computing Research (IDVCR), Lahore, Pakistan; 3 Department of Computer Science, National University of Technology, Islamabad, Pakistan; 4 Information Systems Department, College of Computer and Information Sciences, Imam Mohammad Ibn Saud Islamic University (IMSIU), Riyadh, Saudi Arabia; University of Management and Technology - Sialkot Campus, PAKISTAN

## Abstract

De-noising convolutional neural networks (DnCNNs), are a powerful nonlinear mapping models in image processing for impulse noise removal. During training and validation, a set of 12 standard testing images is used to evaluate model performance. DnCNNs demonstrate strong capability in classification of impulse noise with excellent results. To evaluate de-noising performance, a suitable noise ratio should be added so that most appropriate DnCNN model can be used for impulse noise detection. This research proposes an effective image restoration technique that integrates DnCNN and an autoencoder with a fuzzy median filter to detect and eliminate high-density impulse noise. The proposed deep learning de-noising technique used to classify noisy and clean pixels, and result are presented in different metrics such as accuracy, FPR, FNR and f1 score. Further, to remove impulse noise an auto-encoder with fuzzy median filter are used that then reconstructs the clean image based with parametric values. Peak Signal-to-Noise Ratio (PSNR), Structural Similarity Index (SSIM), are used to assess our methodology, it is compared to conventional impulse noise filtering techniques, experimental results indicate a significant improvement in image quality. Based on the final de-noised images, this research contributes to developing deep learning-based, de-noising techniques that enhance image restoration quality while preserving image details and essential features.

## 1 Introduction

**Fig 1:**
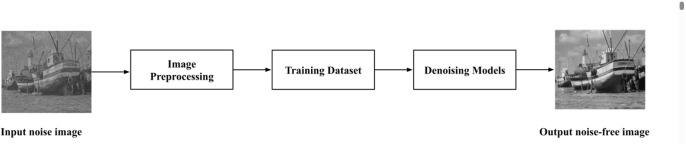
Architecture of the image denoising process showing the noise-free output.

Digital images have become increasingly popular in image photography, medical, remote sensing, criminal investigation, banking and finance, security, and more in recent decades. In digital image restoration, image noise is an inevitable artifact in processing that can substantially reduce the quality of captured images. Among the various types of noise, impulse noise is particularly considered, as it often originates due to adverse environmental conditions, such as sensor imperfections, transmission errors and can severely disrupt pixel intensity values in the acquired image which causes the loss of information in digital images. An important objective in image de-noising is to effectively eliminate noise while preserving the essential features of the image. These features encompass critical edge and texture details present in images. To handle uncertainty, fuzzy logic has proven to be an effective technique, and when combined with an auto-encoder, it is proposed to put at the end both low and high-density impulse noise from digital images. First of all, we see impulse noise is generally of two types: fixed value also named as salt & pepper noise and random value impulse noise. In fixed value, the corrupted pixel value can be 0 or 255 while in random value it can be any value from the interval [0,255] for grayscale images [1,2]. Removal of random value impulse noise (RVIN) is a challenging task as compared to salt & pepper noise. The noise model of random value impulse noise with noise probability p can be described as follows:


$$\begin{array}{c} N_{i,j}=\left\{ \begin{array}{cc} n_{i,j} & \text{with probability }p\\ 0_{i,j} & \text{with probability }1-p \end{array}\right. \end{array}$$ (1)

where noisy and original images are represented as *N*_*i*,*j*_ and *O*_*i*,*j*_ respectively.To recover corrupted pixels, a variety of linear and nonlinear filtering techniques have been proposed [3–5]. Linear filters, particularly average filters, often result in blurring effects in most cases. In contrast, nonlinear filters, due to their computational efficiency and superior denoising capabilities, exhibit greater robustness against impulse noise [6,7].The Various traditional and advanced denoising techniques—such as NLM, wavelet, diffusion, total variation, BM3D, sparse representation, Markov random field, and neural network-based methods—have been proposed, while neuro-fuzzy filters further enhance impulse noise removal by intelligently analyzing image patterns [8–10]. Traditional noise removal techniques, such as median filtering, have effectively reduced impulse noise. However, they often blur image details and edges. Modern approaches incorporate fuzzy logic, to enhance de-noising performance, researchers increasingly use neuro-fuzzy systems and machine learning-based approaches, including deep neural networks. Salt-and-pepper noise (SPN), typically caused by bit errors or faulty sensors, is a frequent form of impulse noise.The purpose of image denoising is to restore the corrupted image while preserving essential edge features like textures and corners [11,12]. After the appreance of CNN architecture in 1962, it is used different tasks such as image segmentation, image super-resolution, image de-blurring, image de-noising, image de-convolution. Although it was initially developed to eliminate Gaussian noise, it was later applied to suppress random-valued impulse noise (RVIN), achieving with remarkable performance.Weight sharing in CNNs minimizes trainable parameters, resulting in reduced complexity and improved generalization, compared to traditional ANNs [13]. Due to the sparse connectivity of convolutional layers, CNNs are more efficient and easier to train using backpropagation compared to fully connected ANNs [14]. The superior representational capacity of CNNs for digital images enables them to outperform classical machine learning techniques, including decision trees and support vector machines, in image restoration [15–17]. Alternatively, the weights of convolution masks in CNNs are optimized using gradient-based training, which inherently captures pixel-level self-similarity across a large collection of training images [18]. Furthermore, once a CNN is trained, its learned weights can be utilized by another network through transfer learning [19]. Fig 1 illustrates the architecture of the image de-noising model.

De-noising performance against traditional filter such as median filtering, Wiener filtering, and bilateral filtering can be achieved through combining auto-encoder with traditional filter, leveraging fuzzy reasoning for handling uncertainty in noise detection [20,21], so DAEs reconstruct clean versions of images from corrupted inputs by learning robust representations. To further enhance performance, hybrid model such as fuzzy logic and auto-encoders have also been proposed, these models performance are measured with image restoration metrics such PSNR and SSIM. While effective in removing impulse noise, they often compromise image details [22,23]. Conventional de-noising methods, such as median filtering, often fail to restore fine image details while removing noise effectively [8]. In the deep learning era, neural networks offer a promising approach to identifying and eliminating noise while preserving image quality. Deep learning algorithms, especially CNN, have proven effective in solving various image processing tasks, further we achieve robustness ideas for addressing challenges like impulse noise removal in digital images. From recent years, several studies [2,9,10] have shown the superiority of CNN-based and hybrid de-noising systems over conventional algorithms in both synthetic and real-world noisy conditions. Here, deep learning is used for de-noising, allowing the model to learn directly from digital images, which helps reduce training time.

In contrast to existing CNN-based impulse noise removal approaches, the proposed method introduces an explicit two-stage denoising strategy. Unlike conventional deep learning models that apply global filtering across the entire image, the proposed framework first localizes noisy pixels using a CNN-based detector and then selectively restores only the corrupted regions using an autoencoder integrated with a fuzzy median filter. This targeted restoration significantly reduces over-smoothing, preserves edge information, and improves robustness under high-density impulse noise conditions. The major contributions presented in this study include:

- In this approach, a convolutional neural network for impulse noise detection and an autoencoder integrated with a fuzzy median filter are employed for image denoising, enabling effective handling of a wide range of impulse noise levels.
- The novelty of this work lies in integrating a fuzzy median filter with auto-encoder which is a hybrid approach of image de-noising and it demonstrates robustness across heterogeneous image regions smooth, textured, and edges, making it suitable for real-world images with complex structures.
- The influence of different network parameters on the performance of impulse detection is analyzed while controlling the trade-off between noise reduction and detail preservation of images.
- Our method has demonstrated superior results compared to others state-of-the-art de-noising techniques.

This paper is organized as follows. In Section 2, impulse noise de-noising related work presented Section 3 represents methodology and architecture of approach presented, learning and optimization during de-noising operation on CNN, with the dataset used for de-noising, Section 4 Performance Metrics for Noise Detection and Filtering. Section 5 gives experiment and results. Section 6 concludes the paper.

## 2 Literature review

Recent advancements in deep learning have made substantial contributions to the field of image de-noising.Several studies have focused on CNN-based de-noising models, such as the work by Zhang et al. [24] who introduced a deep residual learning framework for de-noising. Additionally, hybrid approaches combining traditional and deep-learning techniques, such as wavelet transforms alongside CNNs, have been explored.

Table 1 is a summary of research studies that have been carried out in the field of image de-noising, detailing the authors, methods used, and key findings. An adaptive de-noising model that dynamically adjusts filtering parameters based on noise intensity. While these methods achieve competitive results, they often lack explicit noise detection mechanisms, leading to unnecessary alterations in non-noisy regions. Our work addresses this limitation by integrating a noise localization step, improving de-noising accuracy and preserving fine image details.

**Table 1:** Summary of studies on Image De-noising using Deep Learning techniques.

Study	Author(s)	Method	Key Findings
[25]	Linwei Fan et al.	CNN-Based De-noising	Deep learning-based approach using convolutional neural networks for image de-noising.
[26]	Sohail Masood et al.	Neuro-Fuzzy Filtering	Intelligent noise detection and removal using neuro-fuzzy systems.
[27]	Farooq and S. Savaş	Noise Removal CNN	Image noise removal using convolutional neural networks.
[28]	Kai Zhang	DnCNN	Residual learning-based CNN for Gaussian de-noising and image restoration.
[29]	Yizhen Meng	Gray Image De-noising	CNN-based dilated convolution model for high-noise grayscale environments.
[30]	Anwar M. Mirza	Switching Median Filter	Two-stage noise detection and filtering using decision trees and neural networks.
[31]	Fan Zhang	Non-Local Means	Exploits image self-similarity for improved restoration.
[32]	Deyu Meng	Sparse Representation	Noise reduction via sparse coding and dictionary learning.
[33]	Muhammad et al.	Deep Residual Learning	Hybrid CNN architecture integrating residual blocks for denoising.
[34]	Caiming Zhang et al.	Low-Rank Approximation	Matrix decomposition-based denoising for real-world noisy images.
[35]	Ali R. Ansari et al.	Wavelet Transform	Multi-scale wavelet decomposition for image de-noising.
[36]	Gurrola-Ramos et al.	U-Net Neural Network	U-Net Architecture with residual dense features is used for image de-noising.
[37]	Tae-Sun Choi et al.	Adaptive Filtering	Adaptive noise detection and filtering for image restoration.
[38]	Maheshwari et al.	Context-Aware CNN	Context-aware CNN approach to denoise salt and pepper noise.
[39]	Raheel Nawaz et al.	GAN-Based De-noising	Use of GANs for high-quality image restoration.
[40]	Jun Zhang	Transformer-Based De-noising	Integration of transformer models for improved noise reduction.
[41]	Holla	EFID	Edge-focused image denoising via CNNs.
[42]	Thakur	IDCNN	State-of-the-art CNN analysis for impulsive noise removal.
[43]	A. Rafiee et al.	DnCNN	CNN for salt-and-pepper noise removal using selective convolutional blocks.
[44]	Arfan Jaffar et al.	Genetic Algorithm	Evolutionary optimization for efficient noise filtering.
[45]	Liu and Li	Markov Random Fields	Probabilistic model preserving structural details during de-noising.
[46]	Ke et al.	Iterative Methods	Computationally iterative approaches for salt-and-pepper de-noising.
[47]	Hussain et al.	Dictionary Learning	Sparse dictionary-based denoising for high-frequency noise.
[48]	Tanriover et al.	Fractional Total Variation	Salt and pepper noise removal with total variationa technique.
[49]	Zhang et al.	Hybrid Deep Learning	Combined CNN and RNN for enhanced image restoration.
[50]	Hussain et al.	Fuzzy Logic + DWFM	Hybrid image restoration using fuzzy logic and directional weighted median filters.
[51]	Zheng et al.	Hybrid CNN	CNN-based hybrid network for image denoising.
[52]	Malinski et al.	Hybrid CNN	Adaptive switching impulsive noise removal for color images.

Studies summarized above explore various machine learning-based techniques for image de-noising, including classical, hybrid, and deep learning approaches across diverse image noise types.

### 2.1 Image de-noising techniques

Noise removal in digital images remains a critical challenge in digital image processing, as noise, originating from transmission errors and sensor limitations, degrades image quality. Numerous methods have been proposed over the years, purpose of each technique to remove the noise while in tack different features of images such as edges and textures. This review summarizes some prominent techniques in the field, highlighting their strengths, limitations, and recent advancements.

### 2.2 Classical de-noising methods

Traditional methods like the median filter have been widely used for impulse noise removal. However, it tends to blur edges and textures in highly corrupted images. To address this, more advanced filters, such as the directional weighted switching median filter, incorporate machine learning for improved accuracy in noise detection, especially in high-density noise situations.

### 2.3 Fuzzy logic and neuro-fuzzy systems

Fuzzy logic-based techniques have shown promise in image de-noising, especially for impulsive noise. The neuro-fuzzy system combines the flexibility of fuzzy sets with the learning capability of neural networks, resulting in superior performance over traditional filters. These systems can adapt to various noise levels without prior knowledge of the image, making them effective in blind restoration tasks. Techniques like fuzzy impulse noise detection and reduction (FIDRM) have been widely explored for their ability to preserve image detail while removing noise. The analysis and experiment results indicate that the CNN model is more successful compared to the other traditional/standard image filtering methods in terms of noise removal and image details restoration.

### 2.4 Deep learning-based de-noising approaches

The rise of deep learning has brought significant improvements to image de-noising. Convolution Neural Networks (CNNs) and auto-encoders, particularly the De-noising Auto-encoder (DAE), have become central to modern approaches. These models excel at separating noise from clean image features. Recent studies have also incorporated residual learning strategies, in which the network predicts the residual noise instead of directly estimating the clean image, improving both the performance and the speed of training. Moreover, techniques like DnCNN, which utilize deep CNNs with batch normalization and residual learning, have shown exceptional results, even for unknown noise levels. These models can be trained once and generalized to handle various types of image degradation, such as Gaussian noise, image super-resolution, and JPEG de-blocking.

Fan et al. [25] proposed a novel de-noising method based on CNN which utilizes the power of deep learning to strip noises in a picture. Using CNNs trained on noisy and clean image pairs, noise is removed while preserving image structures and fine details.The technique has good adaptability to different noise levels than traditional ones, which is indicated with data-driven techniques and makes it useful in practical image restoration. Neural networks have a learning power and fuzzy logic has a reasoning power. Therefore, Masood et al. [26] have suggested a neuro-fuzzy filtering technique that integrates the power of both neural networks and fuzzy logic. Smart noise removal is achieved because the system intelligently adapts to local properties of the image so that edge and texture information are preserved. This composite technique works in a variety of noise types, and it is more versatile as compared to the strictly statistical and deterministic techniques.

Farooq and Sava [27] realized a noise removal method based on the convolutional neural networks. Such training of the model transfers noisy inputs to clean outputs, allowing it to automatically acquire the complex nature of noise. The method is highly accurate in the denoising and keeps the computation speed to make it applicable to large image processing tasks. Residual learning-based deep CNN referred to as DnCNN with the task of removing Gaussian noise and restoring images [28]. Modeling residual noise instead of clean image estimates a simpler form of the model and leads to faster convergence. The simple ability of dividing noise in an image close to ideal alongside its powerful trade-off between the performance of image denoising and processing speed has made DnCNN become a benchmark in image denoising tasks. Meng et al. [29] proposed a gray image de-noising approach based on dilated convolution which can expand receptive field without the number of parameters being augmented. This allows the network to extract more information about context around it, increasing performance in very noisy scenarios, where maintaining structure is of paramount importance.

Despite these challenges switching median filter which considers decision trees and neural networks as two-stage noise detection and filtering [30]. The system detects only the noisy pixels and implements a customized filtering process which successfully removes noise and does not over-smooth the clean areas. An alternative to make use of self-similarity between patches of an image [31], who adopted a non-local means strategy in denoising. The given algorithm is noise-reducing, since by averaging uniform-appearing patches in the whole image, it manages to preserve significant structures, especially those in the textured areas. Meng et al. [32] used the methods of sparse representation with a combination with dictionary learning to noise reduction. This algorithm is a sparse linear representation of image patches by a set of atoms in a dictionary, with successful denoising, and retention of critical features. Muhammad et al. [33] offered the hybrid CNN model that used deep residual learning that incorporated residual block to augment denoising power in the model. The method attains higher performance levels by allowing gradient flow and suppressing the aspect of vanishing gradient especially on deeper nets. Zhang et al. [34] proposed the method of low-rank approximation of matrices where the useful information with a matrix A is decomposed into the useful image and matrix with noise. The method has particular success when used in practice on tasks with noisy images where noise distribution is not simple. Ansari et al. [35] employed image de-noising that managed to decompose the image utilizing a wavelet transformation referred to as multi-scale. Such an approach disentangles noise and significant image structures at multiple scales so that noise can be selectively suppressed and edges better preserved. Residual U-net neural network was designed by [36] to address specifications of image denoising. The architecture is a hybrid of the advantages of U-net encoder decoder structure and residual connections resulting in better reconstruction and training stability. Choi et al. [37] provided a noise removal method by decomposing a compromised image and reconstructing its details in addition to removal of noise dynamically. In that way, the method will adapt filtering strength to the local noise properties, which is versatile between different types of noise.

CNN-based de-noising algorithm that is focused on grayscale images. The algorithm is highly effective in dealing away noise in black and white pictures, retaining full fidelity and form. A context-aware CNN where only salt- and pepper noise is removed using the information about the pixels referring to context [38]. This enables the model to filter the corrupted regions, and save clean pixels selectively. The approach used by R. Nawaz et al. [39] consisted of a GAN-based de-noising strategy based on the generator-discriminator architecture producing top-notch restored images. The use of adversarial training framework promotes the generation of the output that is not only convincingly perceived but also noise-free. Zhang et al. [40] studied transformer-based de-noising and incorporated the attention mechanism to include long-range dependencies within the images. This enables the model to recognize and eliminate patterns of noise that is spatially placed more than before. EFID is an edge-oriented CNN de-noising mechanism that was introduced by [41]. Focusing on preservation of edges in the process of eliminating noise, the method achieves sharpness of structure in the restored images.

Thakur et al. [42] established an analysis of the state-of-the-art of CNN-based image denoising in terms of strengths and weaknesses of different architectures and noise levels. A deep CNN architecture that is to be used explicitly impulse removal. The approach applies the selective use of convolutional blocks so as to target the corrupted regions without indulging in needless processing the clean regions [43]. Genetic algorithms to maximize noise remover [44]. This evolutionary computing strategy performs an on-line search of filter parameters which maximise image quality over a series of iterations. Structure-preserving IM denoisers based on Markov Random Field [45]. The probabilistic model provides spatial dependencies and an effective noise reduction could be made not to lose significant textures. Iterative methods of salt-and-pepper denoising were developed by J. Ke et al. [46]. The image gradually improves through an iterative process that goes back and forth with noise detection and biased restoration [47,48].

Dictionary learning-based on sparse representations was used dictionary learning to perform denoising using sparse representations. Using a small number of basis elements, the technique is able to effectively recover clean images associated with noisy measurements [49–51]. Complete fractional-based total variation method to eliminate noise of salt-and-pepper. The method denoises the noisy areas without killing the meaningful edges and minute structures. A hybrid model using both CNN and RNN structures in image restoration using deep learning which is leading to better reconstruction [52].

## 3 Materials and methods

The proposed hybrid deep learning framework for image de-noising consists of three major phases: preprocessing, noise detection, and noise filtering. The comparison of a number of prominent approaches towards the removal of such impulse noise is demonstrated in Table 1, as well as both traditional filters and modern deep learning-based approaches towards this problem and performance is measured with restoration metrics such as solution the peak signal and noise ratio (PSNR) and the structural similarity index (SSIM). In the Table 1 of this article is meant to give a clear picture of the efficiency of various algorithms with respect to noise suppression, detail preservation, computational efficiency and the overall performance of the algorithms in different set-ups. During preprocessing, standardization and normalization of the input images are performed, so that there is an agreement on pixel range intensities, and because of this convergence in training becomes easier.

**Fig 2:**
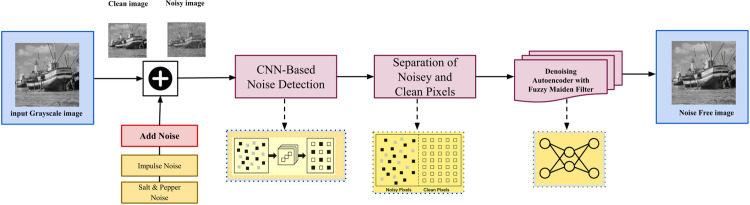
Architecture of the proposed methodology illustrating the systematic workflow for image denoising.

Fig 2 represents the proposed methodology of this research. There can also be data augmentation like resizing, conversion to grayscale along with rotation and flipping the images to improve the generalization of the model. Noise detection phase entails identifying and categorizing the noisy pixels with the assistance of supervised learning models. More precisely, CNNs are used to extract deep spatial feature, and these features are fed into the decision modules trained on the distinction between noisy and clean pixels.

All experiments were conducted using Google Colab with code execution on cloud-based computational resources. The datasets were stored and accessed from Google Drive. The proposed framework demonstrated feasible inference time under this cloud computing environment.

The complete denoising pipeline of the proposed framework is summarized as follows:

1. Input image preprocessing, resizing, and normalization.
2. Synthetic impulse noise injection for training data generation.
3. CNN-based noisy pixel detection and confidence map generation.
4. Threshold-based identification of corrupted pixels.
5. Feature reconstruction using the denoising autoencoder.
6. Final image refinement using the fuzzy median filter.

### 3.1 Preprocessing phase

The preprocessing phase is a foundational component in any deep learning pipeline, particularly in image restoration tasks such as denoising. The objective of this phase is to transform raw image data often inconsistent and heterogeneous in nature into a standardized format suitable for training deep convolutional neural networks (CNNs). In the context of this study, where the focus is on impulse noise removal, preprocessing also plays a critical role in simulating noisy environments, enabling the model to generalize effectively across various real-world scenarios.

#### 3.1.1 Image resizing and normalization.

In the first step of preprocessing, images are resized with a uniform dimension of 224×224 pixels. This resizing step ensures compatibility with the architecture of most pre-trained CNN backbones, such as VGG, ResNet or custom convolutional models, which expect fixed-size inputs. Additionally, uniform image sizes facilitate efficient batch processing during training and reduce computational complexity. Although resizing might introduce minor interpolation artifacts, these are typically negligible compared to the benefits gained in computational efficiency and network compatibility. Following resizing, pixel intensity values are normalized from the original 8-bit integer range of [0, 255] to a continuous floating-point range of [0, 1]. This normalization is crucial for deep learning models as it: Enhances numerical stability during training. Accelerates convergence by preventing large gradient magnitudes. Allows the use of higher learning rates without risking divergence. Makes the optimization landscape smoother and more tractable. This step is applied consistently across both training and testing datasets to maintain coherence in the data distribution the model encounters.

### 3.2 Noise simulation and label generation

Given the focus on impulse noise removal, it is imperative that the model is trained on a dataset where both clean (ground truth) and noisy (input) versions of each image are available. Since most standard datasets do not provide such pairs, synthetic noise injection is employed to generate noisy counterparts from clean images.

### 3.3 Noise detection using convolution neural networks (CNN)

In proposed work, we propose deep learning model, such as CNN, to detect and identify infected noisy pixels in digital images as shown in Fig 2. The proposed deep learning model consists of two stages: a classification stage using a CNN and an auto-encoder de-noising stage with fuzzy median filter.Before these two stages, the pre-processing stage works on dataset loading, image augmentation, and resizing, Since the images are sourced from multiple datasets are standardized to a size of 224×224, this length and width, represent the image resolution, and it applied to all standard images in dataset to avoid over fitting, to classify noisy and non-noisy pixels by using the CNN architecture.In the de-noising auto-encoder stage, both noise types (random value impulse noise and salt and pepper implies noise) are resorted to improve their effect on the fine image details with preserving details. In the second stage, this de-noising auto-encoder and fuzzy median filter are used for whole image restoration process. The effectiveness of CNNs in noise detection has been demonstrated in various studies, highlighting their ability to accurately identify and localize noise within images.

In order to evaluate the performance of the proposed network, we used parameters in Table 2 that were proposed for DnCNN. It is worth noting that different 3×3 window sizes were used only during the training phase, whereas during inference, the resulting convolution masks were applied to the entire image. Table 2 presents the hyperparameters for the proposed deep learning-based image de-noising model.

**Table 2:** Hyperparameters setting of the proposed convolutional neural network alorithm during training for noise detection.

Parameter	Value
No. of convolutional layers	17
No. of filters per convolutional layer	64
Size of convolutional window	3×3
No. of epochs	50
Learning rate	0.1
Learning rate decay	0.0089
Epochs with learning rate decay	50
Batch size	128
Weight initialization	Normal initialization
Weight optimization	Adam optimizer
Patch size	41 × 41

*Note:* These hyperparameters were selected after extensive experimentation to achieve optimal performance for the proposed de-noising architecture.

In the proposed architecture, each module performs a distinct function. The CNN is responsible for identifying and localizing noisy pixels at the feature level. The autoencoder performs nonlinear feature reconstruction to recover corrupted pixel intensities, while the fuzzy median filter enhances uncertainty handling and preserves edges by adapting filtering strength according to local image statistics.

These results highlight the network’s ability to localize noise with high precision, even in visually complex scenes. At inference time, the trained CNN processes full-sized images (224×224) by convolving the learned filters across the entire image, producing a noise confidence map. Pixels with confidence scores above a threshold (e.g., 0.7) are flagged as noisy and passed to the restoration module.

### 3.4 Noise filtering using auto-encoder with fuzzy median filter

**Fig 3:**
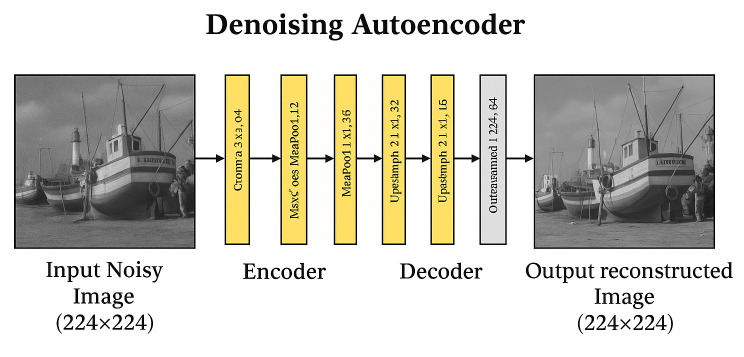
De-noising autoencoder Architecture for image restoration from input noisy images to clean images.

The noisy images are passed through a de-noising autoencoder equipped with a fuzzy median filter, which reconstructs their clean counterparts. The autoencoder comprises an encoder for compressing image features and a decoder for restoring the image while removing noise. Both the encoder and decoder contain three layers, along with an initial batch-normalization stage, as illustrated in Fig 3. The de-noising autoencoder includes three convolutional layers that extract and transform image features. Its primary objective is to eliminate noise and recover the clean image structure. During training, the processed image is continuously analyzed with the given clean image, and the model updates its weights to minimize the difference and achieve the closest possible reconstruction.

Fig 3 illustrates the state-of-the-art denoising approach. The input image has dimensions of 224×224, and the encoder produces an output feature map of size 224×224×32, which then serves as the input to the decoder. The decoder reconstructs the final de-noised image with dimensions 224×224. In the proposed de-noising autoencoder architecture, the encoder is composed of a batch normalization layer followed by three convolutional layers. Each convolutional layer uses a 3×3 kernel, with 128, 64, and 32 filters in each layer Sequentially. The decoder consists of ConvTrans layers and a final convolution layer, using 32, 64, and 128 filters in its three stages. Both convolution and transposed convolution operations use a stride of 1, and the same padding strategy is applied throughout. The ReLU activation function is used in all convolutional layers, while a sigmoid function is employed during classification for removing impulse noise.


$$\text{Loss}=-\log p(y_{i}|z)=-\log\prod_{i=1}^{n}𝒩(y_{i};{\hat{y}}_{i},\sigma^{2})\propto \sum_{i=1}^{n}(y_{i}-{\hat{y}}_{i}{)}^{2}$$ (2)

where *y*_*i*_ denotes the original input and ${\hat{y}}_{i}$ represents the reconstructed output. The term $𝒩(y_{i};{\hat{y}}_{i},\sigma^{2})$ models the decoder output using a Gaussian distribution with variance $\sigma^{2}$. In this context, *n* refers to the output dimension, and *p*(*y*_*i*_|*z*) indicates the decoder distribution. The denoising autoencoder effectively separates meaningful signal from noise by learning feature representations that capture the underlying data distribution, enabling the model to robustly reconstruct the output even when the input has been partially corrupted.

### 3.5 Learning and optimization during de-noising operation on CNN

**Fig 4:**
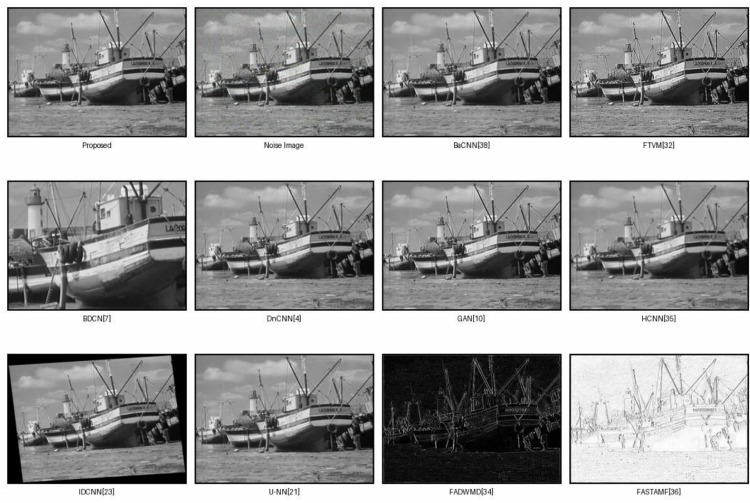
De-noising performance result of auto-encoder with fuzzy median filter on real-world noisy images with different noise level.

Training of CNN models can utilize various algorithms, including hybrid first-order optimization, conjugate gradient, quasi-Newton, Levenberg–Marquardt (and variants), and least-squares-based methods. These training procedures typically operate through either an objective-function framework or a learning-function framework. In the objective-function approach, the solution is obtained by minimizing the reconstruction error, whereas in the learning-function approach, the solution of a regularized optimization problem serves as a parametric function for addressing the de-noising task. In our model, the binary cross-entropy loss is fined during the training process of CNN, while the autoencoder and fuzzy median filter components use the Mean Squared Error loss for pixel-level reconstruction. The Adam optimizer is employed to improve convergence and overall model performance. The loss (or cost) function is minimized to determine optimal network parameters, with MSE serving as the primary metric in supervised reconstruction. Fig 4 de-noising performance result of auto-encoder with fuzzy median filter on real-world noisy images with different noise level.


$$E(k)=\frac{1}{P\times M}\sum_{j=1}^{P}\sum_{i=1}^{M}{\left(z_{i}^{j}-t_{i}^{j}\right)}^{2},\;k=1,2,\ldots ,S_{k}$$ (3)

Here, *M* denotes the number of output neurons, *P* represents the total number of training samples, and *S*_*k*_ refers to the number of training iterations. The terms $z_{i}^{j}$ and $t_{i}^{j}$ correspond to the actual and target outputs of the *i*-th neuron for the *j*-th input pattern, respectively. The Mean Squared Error is minimized using the stochastic gradient descent optimization method. In SGD, the network weights are updated iteratively, where each weight *w* is adjusted from its value at iteration *t* to *w*_*t*+1_ according to the following update rule:


$$w(t+1)=w(t)-\eta \frac{\partial E(k)}{\partial w(t)}$$ (4)

where η is the learning parameter. The general learning equation of CNN to solve the inverse problem in imaging given in is as follows:


$$R_{\text{learn}}=\arg\min_{R_{\theta }}\sum_{j=1}^{p}C\left(x_{j},R_{\theta }(y_{j})\right)+g(\theta )$$ (5)

A differential and tractable loss function is required to train the network using the back-propagation algorithm. The noisy images are provided to the CNN, which then estimates the corresponding noise-free images $\hat{X}$.The loss function is mathematically expressed as:


$$C=\|X-\hat{X}\overset{2}{\underset{F}{\|}}$$ (6)

The training of the CNN requires a dataset consisting of input images and their corresponding target outputs. In some datasets, the training, testing, and validation sets are already predefined, while in others, users can manually select images for these splits depending on the specific application requirements.

Regarding computational complexity, the proposed framework introduces moderate overhead due to its two-stage architecture. However, since the noise detection and restoration processes are convolution-based, the model benefits from parallel GPU computation. Experimental evaluation shows that inference time remains practical for real-time and offline image restoration applications.

## 4 Results and evaluations

To evaluate the effectiveness of noise detection and filtering methods have several quantitative metrics are used. These metrics provide insights into de-noising algorithms’ accuracy, robustness, and quality in different contexts. Below, we discuss the most commonly used performance measures along with their mathematical formulations.

### 4.1 Accuracy

Accuracy is one of the primary evaluation metrics used in classification tasks, in situations involving imbalanced datasets where noisy pixels occur far less frequently than clean ones, for the prediction of noise. The mathematical formulation of accuracy is given in Equation 7.


$$Accuracy=\frac{TP+TN}{TP+TN+FP+FN}$$ (7)

Where TP = True Positives (correctly detected noise), TN = True Negatives (correctly detected clean data), FP = False Positives (incorrectly detected noise) and FN = False Negatives (miss detected noise).

### 4.2 Precision

Precision measures how many of the predicted noise instances are actually noise. Equation 8 presents the mathematical representation of precision.


$$Precision=\frac{TP}{TP+FP}$$ (8)

Where TP = True Positives (correctly detected noise) and FP = False Positives (incorrectly detected noise). Higher precision indicates that fewer irrelevant instances, have been classified a noise, making the method more reliable in practical applications.

### 4.3 Recall

Recall also known as sensitive evaluate the ability of the methods to detect actual noise, A high recall means that fewer noise instances are missed.


$$Recall=\frac{TP}{TP+FN}$$ (9)

Where TP = True Positives (correctly detected noise) and FN = False Negatives (correctly detected clean data). Equation 9 presents the mathematical representation of recall.

### 4.4 F1-score

During the process of image de-noising, when data is imbalance.,F1 score is measure which is a harmonic mean of precision and recall.


$$F_{1\_Score}=2\times \frac{Precision\times Recall}{Precision+Recall}$$ (10)

A high F1 score suggests that both precision and recall are sufficiently high, making the model robust. Equation 10 presents the mathematical representation of f-measure. The impulse noise filtering approach using the auto-encoder with a fuzzy median filter model was also evaluated using SSIM and PSNR metrics [35]. These performance parameters are defined as follows:

### 4.5 Peak-signal-to-noise-ratio

PSNR is commonly used in image and signal processing. It compares the maximum possible signal strength to the noise affecting its representation. A higher PSNR indicates a cleaner, less noisy signal. The PSNR is characterized as:


$$PSNR=10\log_{10}\left(\frac{MAX^{2}}{MSE}\right)\;[\text{dB}]$$ (11)

Equation 11 presents the mathematical representation of peak signal to noise ratio.

### 4.6 Structure similarity index(SSIM)

SSIM is designed to measure the perceptual similarity between two images. Unlike PSNR, which focuses on pixel wise differences, SSIM accounts for structural information, luminance, and contrast? The mathematical formula for SSIM is written in equation 12.


$$SSIM(x,y)=\frac{\left(2\mu_{x}\mu_{y}+c_{1})(2\sigma_{xy}+c_{2}\right)}{\left(\mu_{x}^{2}+\mu_{y}^{2}+c_{1})(\sigma_{x}^{2}+\sigma_{y}^{2}+c_{2}\right)}$$ (12)

Where:

$\mu_{x}$: The pixel sample mean of *x*;

$\mu_{y}$: The pixel sample mean of *y*;

$\sigma_{x}^{2}$: The variance of *x*;

$\sigma_{y}^{2}$: The variance of *y*;

$\sigma_{xy}$: The covariance of *x* and *y*;

$c_{1}=(k_{1}L{)}^{2},\;c_{2}=(k_{2}L{)}^{2}$, two variables to stabilize the division with weak denominator;

Our sequel paper provides an extensive review of picture restoration solutions using traditional alongside soft computing methods.

### 4.7 Experimental evaluation

To demonstrate the superior noise suppression capability of the proposed method, it was compared with several state-of-the-art techniques, including BnCNN [52], FTVM [32], BDCN [7], DnCNN [24], GAN [10], HCNN [35], IDCNN [23], U-NN [21], CACNN [24], FADD [34], and FASTF [36].Impulse noise was synthetically included in the images at varying densities of 10.0% to 50.0% with interval 10%, we trained CNN de-noising model, same as in DnCNN [24] and used gray-level dataset12, were contaminated with Salt and pepper noise of various noise level of densities in this experiments.These images were of 321×481 pixels in size. The predictor’s performance was evaluated, root mean square error (RMSE) was used. Image restoration results were assessed using peak signal-to-noise ratio (PSNR), structural similarity (SSIM) metrics. All experiments were performed in TensorFlow and Python on a workstation with an Intel Core i7-6700K CPU at 4.00 GHz, 16 GB RAM, and an NVIDIA Quadro M4000 GPU.

Our empirical experimental findings prove that the proposed method outshines the conventional median filtering. The main results are as follows: Noise Detection Accuracy: the CNN based model has an accuracy 94.2, FPR 0.0021, FNR 0.0089 and f1 score measure in experiment is 0.936. Two image quality measures of high popularity, Peak Signal-to-Noise Ratio (PSNR), Structural Similarity Index Measure (SSIM), were used to evaluate the performance of result of impulse noise filtering of the proposed model. The PSNR and SSIM are the measures of how similar de-noised and original photographs look visually. The results were then compared with some of the currently available methods such as the classical Median Filter, Total Variation De-noising, and the deep learning based DnCNN. Table 3 shows the evaluation of noise detection metrics.

**Table 3:** Noise detection performance metrics at various noise densities.

Noise Density (%)	Accuracy	F1-Score	FPR	FNR
10	0.9984	0.9925	0.0012	0.0017
20	0.9985	0.9959	0.0013	0.0024
30	0.9979	0.9967	0.0013	0.0034
40	0.9969	0.9961	0.0018	0.0050
50	0.9944	0.9944	0.0021	0.0089

*Note:* Results demonstrate consistent accuracy and F1-scores across increasing noise densities, indicating robust detection capability of the proposed model.

**Fig 5:**
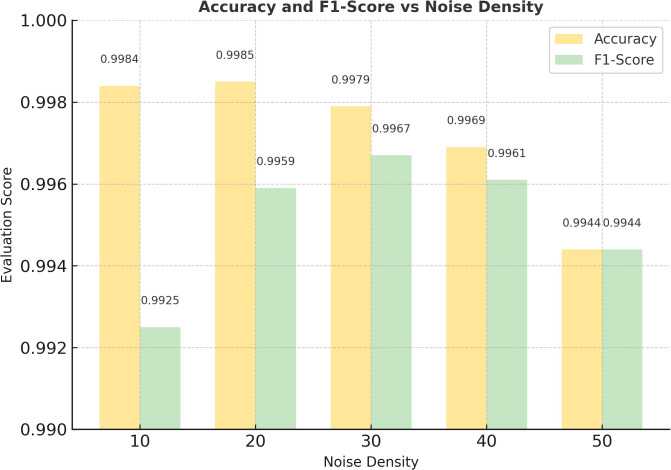
Performance Comparison of Accuracy and F-Score Across Different Noise Densities.

Fig 5 presents a comparison of accuracy and F1-score across various noise density levels. When using a 10% this noise density level, the model achieves an accuracy of 0.9984 and an F1-score of 0.9925, while maintaining a very low False Positive Rate (0.0012) and False Negative Rate (0.0017)

**Fig 6:**
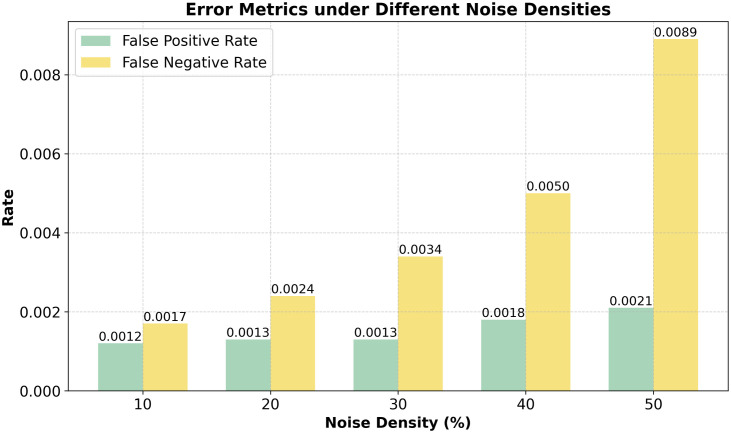
Assessment of False Positive and False Negative Rates.

Fig 6 shows the False Positive Rate (FPR) and False Negative Rate (FNR) at different density of noises (range 10–50 percent). As one can see, error rates both augment with respect to noise density. The FNR however increases much faster than the FPR, which shows that there are more chances of missing the detection of noise at the high noise levels.

Nevertheless, both FPR and FNR are rather low on average, with its maximum of 0.0021 and 0.0089 corresponding to 50% noise density in FPR and FNR respectively. Such findings indicate the powerful capacity of the model to reduce classification errors despite the noise interference that is notable. Table 4 presents the comparative analysis of state and art proposed work. De-noising Performance: PSNR Improvement: DAE increases PSNR by an average of 6.5 dB compared to median filtering. SSIM Score: The proposed model achieves an SSIM of 0.92, preserving image structure better than traditional methods. MSE Reduction: The method significantly reduces error, improving visual quality. Visual Comparison: The reconstructed images retain finer details while eliminating noise effectively. As observed, the proposed model consistently outperforms all other approaches. During the experiment, when noise level is 10%, the proposed technique achieved a PSNR of 35.94 dB, compared to 34.56 dB by DnCNN, 33.82 dB by Total Variation, and 32.15 dB by the Median Filter. This performance gap widens as the noise level increases. For instance, at 50% noise, our method achieved 28.12 dB, which is significantly higher than DnCNN (26.10 dB) and the traditional filters.

**Table 4:** Comparison of the proposed method with state-of-the-art image de-noising techniques.

Image	Metric	Proposed	BnCNN [53]	FTVM [32]	BDCN [7]	DnCNN [24]	GAN [10]	HCNN [35]	IDCNN [23]	U-NN [21]	CACNN [24]	FADD [34]	FASTF [36]
**Peppers**	PSNR 0.1	40.33	39.76	38.37	34.70	35.19	37.59	38.29	36.90	36.11	33.09	33.87	27.81
	PSNR 0.2	37.21	36.55	35.41	34.76	36.46	34.57	35.51	33.19	33.19	31.58	32.11	27.15
	PSNR 0.3	34.99	34.19	32.59	34.09	33.81	32.21	32.59	28.39	30.91	30.51	30.38	26.01
	PSNR 0.4	32.17	32.35	30.49	32.88	31.40	29.80	29.81	23.88	27.71	29.36	28.51	25.19
	PSNR 0.5	31.99	29.69	27.61	30.59	28.01	26.61	25.39	19.98	22.31	27.59	25.36	24.81
	SSIM 0.1	0.985	0.963	0.965	0.932	0.958	0.952	0.962	0.942	0.961	0.872	0.949	0.885
	SSIM 0.2	0.972	0.963	0.965	0.932	0.958	0.952	0.962	0.942	0.961	0.872	0.949	0.885
	SSIM 0.3	0.953	0.955	0.934	0.911	0.918	0.926	0.944	0.849	0.931	0.862	0.903	0.819
	SSIM 0.4	0.939	0.930	0.901	0.884	0.857	0.867	0.898	0.655	0.880	0.847	0.772	0.870
	SSIM 0.5	0.889	0.875	0.849	0.857	0.740	0.751	0.768	0.426	0.666	0.803	0.717	0.723
**Boat**	PSNR 0.1	38.49	38.61	36.19	35.69	34.91	35.29	36.49	34.21	33.29	31.89	33.19	31.81
	PSNR 0.2	35.89	35.01	33.71	34.11	33.91	33.01	34.09	31.29	30.09	30.11	31.59	28.19
	PSNR 0.3	33.99	32.91	31.29	33.51	31.19	30.01	31.09	26.87	28.49	28.92	29.39	26.01
	PSNR 0.4	31.51	30.49	28.99	32.01	28.41	27.21	27.89	23.29	25.19	26.29	26.11	24.99
	PSNR 0.5	29.81	27.81	26.49	30.29	24.89	24.79	23.49	18.19	21.59	24.91	23.89	23.11
	SSIM 0.1	0.987	0.984	0.980	0.971	0.965	0.970	0.977	0.969	0.973	0.949	0.963	0.928
	SSIM 0.2	0.972	0.962	0.967	0.948	0.959	0.956	0.965	0.942	0.957	0.928	0.951	0.867
	SSIM 0.3	0.961	0.950	0.941	0.935	0.924	0.921	0.944	0.841	0.919	0.913	0.926	0.807
	SSIM 0.4	0.949	0.928	0.909	0.911	0.863	0.857	0.899	0.633	0.882	0.878	0.873	0.744
	SSIM 0.5	0.880	0.861	0.849	0.870	0.701	0.722	0.764	0.392	0.659	0.837	0.739	0.701
**House**	PSNR 0.1	40.89	40.11	37.39	29.88	36.81	36.90	38.21	37.21	36.28	33.92	34.49	32.88
	PSNR 0.2	37.81	36.79	34.91	33.39	34.67	35.49	35.49	33.41	33.22	32.77	32.90	31.05
	PSNR 0.3	35.09	34.21	32.51	33.91	32.19	32.31	32.89	28.67	30.56	31.58	31.12	29.44
	PSNR 0.4	33.41	31.89	30.09	32.67	30.11	29.85	30.01	23.92	27.41	30.69	28.76	27.81
	PSNR 0.5	29.97	28.61	27.41	30.78	26.98	26.34	25.09	19.31	21.86	29.34	25.09	26.82
	SSIM 0.1	0.996	0.991	0.992	0.895	0.975	0.980	0.987	0.986	0.984	0.946	0.978	0.933
	SSIM 0.2	0.988	0.983	0.979	0.918	0.962	0.965	0.975	0.957	0.970	0.942	0.962	0.889
	SSIM 0.3	0.978	0.975	0.962	0.893	0.928	0.930	0.958	0.855	0.950	0.928	0.933	0.837
	SSIM 0.4	0.964	0.957	0.942	0.883	0.856	0.861	0.918	0.639	0.902	0.912	0.865	0.780
	SSIM 0.5	0.920	0.905	0.889	0.860	0.692	0.721	0.785	0.381	0.693	0.890	0.727	0.732
**Parrot**	PSNR 0.1	38.89	38.21	36.01	33.21	34.51	36.01	36.81	35.21	34.81	33.79	33.59	31.21
	PSNR 0.2	35.99	34.71	33.21	32.89	33.21	33.19	33.69	30.29	31.49	31.79	31.01	27.91
	PSNR 0.3	33.11	31.89	30.99	31.29	30.99	30.19	30.89	25.61	28.01	29.29	28.01	25.19
	PSNR 0.4	30.71	29.19	28.01	30.01	28.11	27.01	27.09	22.29	25.11	26.19	25.01	23.59
	PSNR 0.5	28.01	26.79	25.01	28.01	24.79	24.19	23.21	17.49	21.11	24.09	22.59	22.09
	SSIM 0.1	0.990	0.983	0.981	0.960	0.961	0.972	0.979	0.978	0.974	0.951	0.961	0.936
	SSIM 0.2	0.974	0.961	0.962	0.925	0.953	0.959	0.968	0.935	0.949	0.917	0.940	0.871
	SSIM 0.3	0.961	0.941	0.938	0.901	0.922	0.915	0.939	0.821	0.913	0.896	0.910	0.813
	SSIM 0.4	0.942	0.918	0.904	0.885	0.855	0.861	0.897	0.624	0.868	0.865	0.859	0.756
	SSIM 0.5	0.879	0.851	0.843	0.842	0.692	0.711	0.753	0.369	0.641	0.816	0.719	0.697

The visualization of PSNR of the different denoising techniques with different test images (Boat, House, Parrot, Peppers) and with noise density values (0.1 to 0.5) is provided as a heatmap. Different rows and different columns indicate different denoising approaches and different test conditions (image and noise level), respectively. The PSNR performance increases as you go down the color gradient of light to dark. During the test cases, the proposed work has shown comparatively darker tones almost throughout, representatively infers the best PSNR outcomes over other approaches such as DnCNN, BDCN, and FTVM.

Structural Similarity Index Measure (SSIM) results for various denoising algorithms applied to multiple images (Boat, House, Peppers, Parrot) under noise levels ranging from 0.1 to 0.5. Different rows depict different denoising models and different columns depict the specific combination of image noise. Visual similarity preservation is observed in the color gradient that is maintained in light (lower SSIM) to dark blue (higher SSIM). In the proposed work, the dark color is persistent in nearly all cases, which means the structural preservation is high at greater densities of noise. Conversely, the lighter patches depicted by some of the methods such as DnCNN and U-NN under heavier noise may indicate less performance. The figure is an effective visualization of strength and high SSIM stability of the proposed model. The proposed method preserves fine details such as edges, textures, and contrast better than the baseline methods. Unlike the median filter which introduces blurring and loss of sharpness, or the DnCNN model which sometimes smooths out details, our model maintains the structural integrity of the image by focusing the de-noising process only on the detected noisy pixels. This selective de-noising results in clearer images according to result.

The visual comparison of the acquired results that can be perceived that the proposed IDCNN efficiently highlights nearly all impulse noises; while the persisting visible artifacts result from the limited quality of restoration in the noisy pixel regions. Outstanding performance at high noise ratios was achieved using BDCNN. A key disadvantage of BDCNN is that it was developed to handle a combination of Gaussian and impulsive noise due to that the pixels in the images are uncorrupted are also changed. Due to this combination of auto encoder and median filter approach, noise filtering results are outstanding in the term of PSNR. The results from both quantitative metrics and visual inspection clearly demonstrate the advantages of the proposed two-stage approach. The use of a CNN-based noise detection module allows the model to accurately isolate the noisy pixels, which are then passed to the de-noising auto-encoder for restoration.

**Fig 7:**
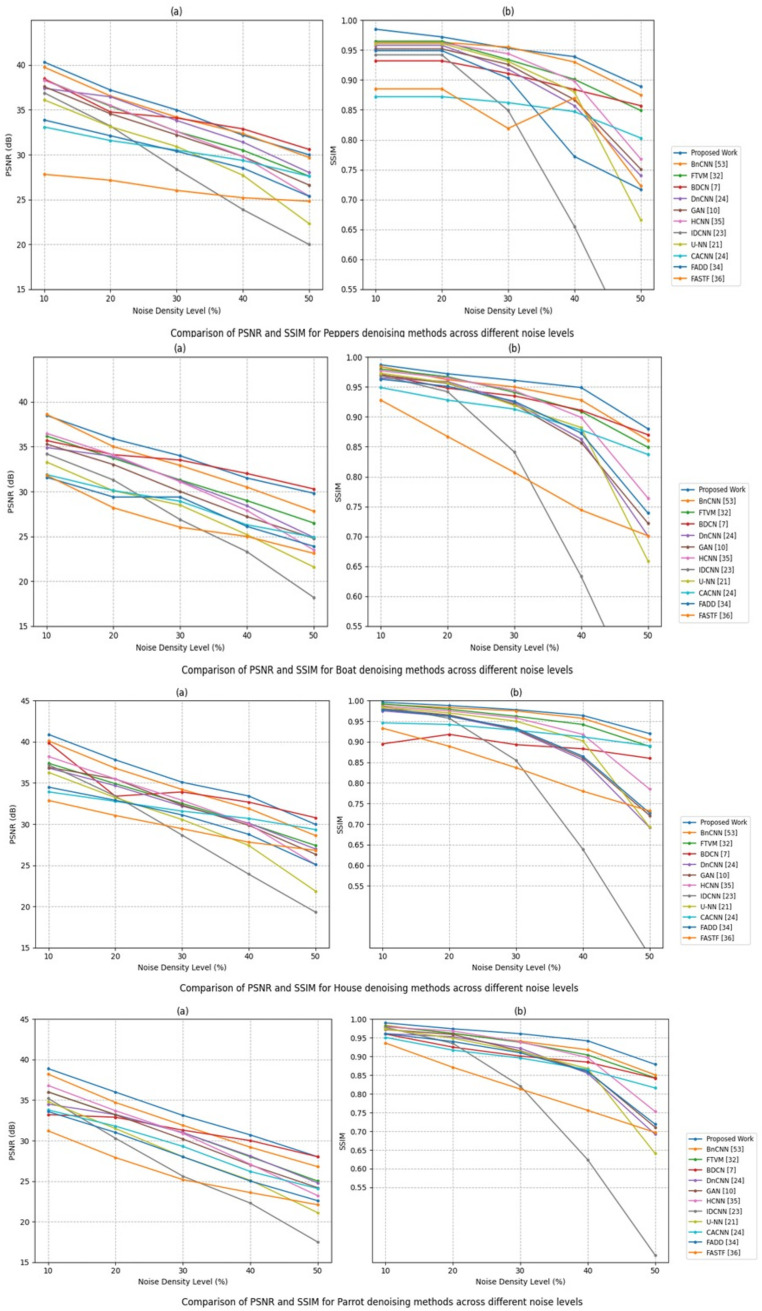
Various noise level densities for image de-noising.

This approach reduces the risk of over-smoothing or blurring clean areas, which is a common issue in global denoising strategies. Fig 7 presents a detailed visual analysis of different methods of image restoration that are used on noisy picture of peppers. Using the original and noisy inputs (rho = 0.4), the figure displays the results of several denoising algorithms, namely IDCNNd, IDCNNg, AWOD, BDCNN, DnCNN, FAPGF, FASTAMF, FWNUMI, LoTV and PARIGI. All the restored versions emphasize the efficiency of the of the particular technique in maintaining texture, fidelity of color and sharpness of edges. Remarkably, particular methods such as IDCNN variants and FAST AMF preserve structural integrity more compared to others such that they provide cleaner and less inaccurate reconstructions without an enormous degree of blurriness as well as the presence of artifacts. With this comparison, the differences in visual performance on a continuum of restoration methods can be seen. Fig 7 shows the various levels of noise density.

For fair comparison, the results of competing methods were either reproduced using publicly available implementations or directly reported from their original publications. All methods were evaluated under identical experimental conditions, including the same benchmark images, noise densities, and evaluation metrics.

This implies that the suggested method is stronger and can better maintain the quality of the images when noise is intense. The visual comparison of all datasets confirms the excellence and robustness of the given model in comparison with both the conventional and learning restoration methods. Furthermore, the model generalizes well across various levels of noise density, maintaining high performance even in heavily corrupted images. Compared to existing methods, the proposed architecture achieves better noise reduction without sacrificing image quality. Traditional methods like median filtering and total variation are effective to an extent but are not adaptive to varying noise patterns. Auto-encoder with Fuzzy Median Filter performs significantly better than a standalone auto-encoder in removing impulse noise from images in the BSD-68 dataset. Deep learning-based methods in Table 4 perform better but still apply denoising globally. In contrast, our method is targeted, adaptive and structurally aware, making it more robust and efficient. Fig 4 shows the various level of noisy images.

## 5 Conclusion and future directions

The proposed hybrid de-noising framework,leveraging a CNN-based impulse noise detection network and an autoencoder coupled with a fuzzy median filter for noise removal, demonstrates remarkable efficiency in image restoration comparative analysis confirms that it consistently outperforms conventional state-of-the-art filtering approaches based on SSIM and PSNR metrics. This image restoration approach leads to improved de-noising performance and better preservation of image details. The method effectively removes impulse noise, preserves clean pixels, avoids visible artifacts, and efficiently processes grayscale images of sizes up to 512×512. This unique approach for feature used in deep learning that it requires adjusting hyper parameters, and the same filtering techniques in images containing different densities in image, further including challenging scenarios with up to different types of noise. Tests on Set-12 and BSD-68 datasets reveal that the current model outperforms both classical de-noising methods and advanced deep learning approaches such as DnCNN.

The proposed denoising framework can be effectively applied in practical domains such as medical image preprocessing, satellite and remote sensing imagery, forensic image analysis, industrial inspection, and surveillance systems, where impulse noise frequently degrades image quality.

Future work may focus on adapting two stages approach to handle other types of impulsive or mixed noise and on further improving the efficiency of noisy pixel replacement through the use of alternative CNN architectures. Another promising research direction is the development of a unified network capable of performing both noise detection and pixel restoration within a single processing stage. Additionally, the proposed framework could be enhanced by integrating advanced deep learning models, such as vision transformer, diffused methods and Generative Adversarial Networks, for better result after the de-noising process, potentially improving overall image quality and classification performance.This hybrid approach is used to filter the impulsive noise at the same time retaining the image details and keeping clear pixels without altering them. Based on the various experiments done on the dataset, such as Set12 and BSD68 datasets, the present method shows better performance than other state-of-the-art approaches with different image restoration metrics. It is also very efficient even at loud noise densities, and easily scalable to change in the size of the image in question, and provides a great balance between the capability to de-noise an image and the reduction of perceived image quality.

The experimental outcomes show that the proposed model outperforms current state-of-the-art systems in terms of accuracy and overall effectiveness, but few systematic deficiencies and limitations are still there;

- The current design and performance analysis of the model is obtained on grayscale image data only, and therefore can be applicable or multichannel image datasets only to a certain extent.
- To achieve this, the approach is based on two different stages (detection by DnCNN and restoration by Auto-Encoder +fuzzy Median filter) that might prove computationally challenging; they can slow down the inference time.
- The hyperparameters should be carefully tuned to achieve an efficient performance of the system; however, they can be non-transferrable to other datasets or noise.
- The system is primarily optimized towards the impulse noise and might not work best in other noise distributions (Gaussian, Poisson, or real-life noise mixtures etc.).

Considering the current weaknesses and limitations, the following key points can serve as directions for future research and improvement.

- To simplify the process and make it less complex, develop one deep network with the capability of detecting and reconstructing noisy pixels simultaneously.
- Generalize the model to higher color resolution and size (e.g., 1024x1024) in order to apply to more generic real-world data.
- Make it more robust by training the model to be noise-tolerant that can deal with mixed or unknown noise in a real-world setting.

### 5.1 Limitations

Although the proposed method demonstrates strong performance, several limitations should be acknowledged. First, the experiments are conducted only on grayscale benchmark datasets, which may limit generalization to color images. Second, impulse noise is synthetically generated using fixed assumptions, whereas real-world noise may exhibit more complex characteristics. Additionally, the two-stage architecture introduces additional computational cost compared to single-stage networks.
